# Supplementary material for: Quantum clocks observe classical and quantum time dilation
Source: Nat Commun. 2020 Oct 23;11:5360. doi: 10.1038/s41467-020-18264-4 (PMC7584645; doi:10.1038/s41467-020-18264-4)
Supplement: Supplementary file 1 — Supplementary Information [file 41467_2020_18264_MOESM1_ESM.pdf]

Supplementary Information: Quantum clocks observe classical and quantum time dilation

Alexander R. H. Smith[1,2] and Mehdi Ahmadi[3]
[1]Department of Physics, Saint Anselm College, Manchester, New Hampshire 03102, USA
[2]Department of Physics and Astronomy, Dartmouth College, Hanover, New Hampshire 03755, USA
[3]Department of Mathematics and Computer Science,
Santa Clara University, Santa Clara, California 95053, USA
(Dated: August 11, 2020)

SUPPLEMENTARY NOTE 1: RECOVERING THE KLEIN-GORDON EQUATION IN THE PAGE-WOOTTTERS FORMALISM

For simplicity, let us consider a single particle situated in Minkowski space, so that the constraint in Eq. (31) of the main text becomes

$$C_H \,|\Psi\rangle\rangle = \Big(\eta^{\mu\nu} P_\mu P_\nu \otimes I_C + I_0 \otimes I_{\rm cm} \otimes \big(m + H_{\rm clock}/c^2\big)^2 c^2\Big) \,|\Psi\rangle\rangle = 0, \tag{1}$$

where $\eta_{\mu\nu}$ denotes the Minkowski metric and we have suppressed the subscript n. Given a physical state satisfying this constraint, Eq. (36) of the main text defines the conditional state of the center-of-mass and internal degrees of freedom of the particle

$$|\psi_S(t,\mathbf{x})\rangle := \big(\langle t|\,\langle \mathbf{x}| \otimes I_C\big)\,|\Psi\rangle\rangle\,,$$

where $x^0\,|t\rangle = t\,|t\rangle$ and $|\mathbf{x}\rangle := |x^1\rangle\,|x^2\rangle\,|x^3\rangle$ with $|x^i\rangle$ denoting an eigenstate of the operator x^i. Now consider the action of the d'Alambertian operator $\Box := \eta^{\mu\nu}\partial_\mu\partial_\nu$ on the conditional state

$$\begin{aligned}
\Box\,|\psi_S(t,\mathbf{x})\rangle &= \eta^{\mu\nu}\partial_\mu\partial_\nu\big(\langle t|\,\langle \mathbf{x}| \otimes I_C\big)\,|\Psi\rangle\rangle \\
&= \eta^{\mu\nu}\partial_\mu\partial_\nu\Big(\langle t_0|\,e^{iP_0 t}\,\langle \mathbf{x}_0|\,e^{iP_i x^i} \otimes I_C\Big)\,|\Psi\rangle\rangle \\
&= \big(\langle t|\,\langle \mathbf{x}| \otimes I_C\big)\big(\eta^{\mu\nu}P_\mu P_\nu \otimes I_C\big)\,|\Psi\rangle\rangle \\
&= -\big(\langle t|\,\langle \mathbf{x}| \otimes I_C\big)\Big[I_0 \otimes I_{\rm cm} \otimes \big(m + H_{\rm clock}/c^2\big)^2 c^2\Big]\,|\Psi\rangle\rangle \\
&= -I_0 \otimes I_{\rm cm} \otimes \big(m + H_{\rm clock}/c^2\big)^2 c^2\,|\psi_S(t,\mathbf{x})\rangle\,,
\end{aligned} \tag{2}$$

where the third equality is obtained using Eq. (1). Upon rearranging Eq. (2) we find that the conditional state satisfies

$$\Big[\Box + \big(m + H_{\rm clock}/c^2\big)^2 c^2\Big]\,|\psi_S(t,\mathbf{x})\rangle = 0, \tag{3}$$

where we have suppressed the identity operators I_0, I_C, and $I_{\rm cm}$. If one supposes $H_{\rm clock}$ vanishes, then Eq. (3) reduces to the usual Klein-Gordon equation.

SUPPLEMENTARY NOTE 2: JUSTIFICATION FOR USING THE PAGE-WOOTTERS FORMALISM

One might question why a more standard formulation of relativistic quantum mechanics was not used. We feel the following excerpt, that has been edited for clarity, from Feynman's 1964 Messenger lectures delivered at Cornell University justifies why one should adopt a plurality of theoretical approaches to describe a given phenomena:

> "Consider two identical theories A and B, which look completely different psychologically and have different ideas in them, but all their consequences are exactly the same. A thing that people often say is how are we going to decide which one is right?
>
> No way! Not by science because both theory A and theory B agree with experiment to the same extent so there is no way to distinguish one from the other. So if two theories, though they may have deeply

very different ideas behind them, can be shown to be mathematically equivalent then people usually say in science that the theories can not be distinguished.

However, theories A and B for psychological reasons, in order to guess new theories, are very far from equivalent because one gives the scientist very different ideas than the other. By putting a theory in a given framework you get an idea of what to change. It may be the case that a simple change in theory A may be a very complicated change in theory B. In other words, although theories A and B are identical before they're changed, there are certain ways of changing one that look natural which don't look natural in the other.

Therefore, psychologically we must keep all the theories in our head and every theoretical physicist that is any good knows six or seven different theoretical representations for exactly the same physics, and knows that they are all equivalent, and that nobody is every going to be able to decide which one is right at that level. But they keep these representations in their head hoping they will give them different ideas for guessing."

In this case, the Page-Wootters formalism suggested to formulate time dilation in terms of the conditional probability distribution in Eq. (12) of the main text.

SUPPLEMENTARY NOTE 3: PROOF OF DESIDERATA OF PHYSICAL CLOCKS THEOREM

The theorem stated in the Results is a summary of well-known results of quantum parameter estimation [1–4]. We summarize here how the two properties of the theorem follow from the covariance properties of the POVM.

The first statement follows from a direct computation of the average of T_{clock} on the state $\rho(\tau)$

$$\begin{aligned}
\langle T_{\text{clock}} \rangle_{\rho(\tau)} &= \int_G d\tau' \; \text{tr}\left[E(\tau')\rho(\tau)\right]\tau' \\
&= \int_G d\tau' \; \text{tr}\left[U^\dagger_{\text{clock}}(\tau)E(\tau')U_{\text{clock}}(\tau)\rho\right]\tau' \\
&= \int_G d\tau' \; \text{tr}\left[E(\tau'-\tau)\rho\right]\tau' \\
&= \int_G d\tau' \; \text{tr}\left[E(\tau')\rho\right](\tau'+\tau) \\
&= \tau,
\end{aligned}$$

where the third equality follows from Eq. (3) of the main text, the fourth equality follows from a change of variables $\tau' \to \tau' - \tau$, and in arriving at the last equality we used the fact that by construction $\langle T_{\text{clock}} \rangle_\rho = 0$.

The second statement follows in a similar manner

$$\begin{aligned}
\langle (\Delta T_{\text{clock}})^2 \rangle_{\rho(\tau)} &= \int_G d\tau' \; \text{tr}\left[E(\tau')\rho(\tau)\right]\tau'^2 - \langle T_{\text{clock}} \rangle^2_{\rho(\tau)} \\
&= \int_G d\tau' \; \text{tr}\left[E(\tau'-\tau)\rho\right]\tau'^2 - \tau^2 \\
&= \int_G d\tau' \; \text{tr}\left[E(\tau')\rho\right](\tau'+\tau)^2 - \tau^2 \\
&= \langle (\Delta T_{\text{clock}})^2 \rangle_\rho.
\end{aligned}$$

[1] Holevo, A. S. *Probabilistic and Statistical Aspects of Quantum Theory*, vol. 1 of *Statistics and Probability* (North-Holland, Amsterdam, 1982).

[2] Braunstein, S. L. & Caves, C. M. Statistical distance and the geometry of quantum states. *Phys. Rev. Lett.* **72**, 3439–3443 (1994).

[3] Braunstein, S. L., Caves, C. M. & Milburn, G. J. Generalized Uncertainty Relations: Theory, Examples, and Lorentz Invariance. *Annals of Physics* **247**, 135–173 (1996).

[4] Wiseman, H. M. & Milburn, G. J. *Quantum Measurement and Control* (Cambridge University Press, Cambridge, UK, 2010).
